# Consequences and adaptation in daily life – patients’ experiences three decades after a nerve injury sustained in adolescence

**DOI:** 10.1186/1471-2474-14-252

**Published:** 2013-08-22

**Authors:** Anette Chemnitz, Lars B Dahlin, Ingela K Carlsson

**Affiliations:** 1 Department of Hand Surgery, Lund University, Skåne University Hospital, S-205 02, Malmö, Sweden

**Keywords:** Peripheral nerve injury, Outcome, Consequences, Adaptation, Adolescence, Qualitative study

## Abstract

**Background:**

To explore the patients’ experiences during the three decades following repair of a nerve injury in the forearm and its consequences for daily life. Strategies that were used to facilitate adaptation were also investigated.

**Methods:**

Fifteen participants with a complete median and/or ulnar nerve injury repaired in the ages from 13–20 years were interviewed using a semi-structured interview guide. The median follow-up time was 31 years (range 23–40). The participants were asked to describe the past and present symptoms of the injured hand, the consequences of the injury for daily life, personal qualities and support from others. In addition, they were asked to describe strategies used to facilitate adaptation. The interviews were subjected to content analysis.

**Results:**

The nerve injury lead to sensory and motor deficits in the injured hand, as well as sensitivity to cold and secondary back problems. Emotional reactions to trauma and symptoms related to post-traumatic stress disorder were described, as well as how they managed to cope with such reactions. There was a noticeable impact on education, leisure, professional or domestic life for some, while others could continue by changing e.g. their performance pattern. The participants’ life roles and relations were also affected. Both emotion- and problem-based strategies were used to manage challenges in daily life.

**Conclusions:**

The present qualitative study can help us to provide the patient with honest and realistic information about what to expect after a nerve injury at forearm level, without eliminating hope. Emotional reactions to trauma should be identified and dealt with. In addition, health-care professionals can promote a variety of coping mechanisms to facilitate daily living for the injured patients.

## Background

Nerve injuries at forearm level may have a variety of serious consequences for the individual and for society. Reduced sensibility, grip function and dexterity as well as cold sensitivity are common, known symptoms [1-5]. However, patients may also be affected psychologically by such an injury. Recent studies have shown high rates of depression after nerve injury in the upper extremity [6] and after severe hand injuries [7-9]. Symptoms of posttraumatic stress disorder are often overlooked, despite the importance of considering the psychological status when caring for patients with hand injuries [7]. In published papers about treatment after a peripheral nerve injury, the focus is mainly on how to enhance motor and sensory recovery [10-12]. Nevertheless, the patients’ past medical history, personality, social and cultural background, occupation and hobbies all need to be considered in order to optimise long term satisfaction in the patient [13].

Recently, we published a long-term follow- up of functional outcome after repair of median and ulnar nerve injuries performed in childhood and adolescence [14]. This retrospective study with a median follow-up of 31 years shows that the nerve injury had had a significant impact on education, leisure activities and choice of profession for the patients injured in adolescence. Such aspects, as well as psychological aspects in the long term, have not been sufficiently considered previously. Our present aim was therefore to explore patients’ experiences of the nerve injury and its consequences for daily life during the three decades following the repair. In addition, we wanted to investigate the personal qualities and strategies that were used to facilitate adaptation.

## Methods

### Participants

These participants were included in a larger study focusing on the epidemiology and the long-term clinical outcome after nerve repair in childhood and adolescence [14]. The results show a significant impact on education, leisure activities and choice of profession only for patients injured in adolescence. In the current interview study, we wanted to explore this group of patients further and purposive sampling was used giving a variation in age at injury, gender, type of nerve injury and whether the dominant or non-dominant hand was injured. The inclusion criteria were as follows: the participants should not suffer from other serious disorders that might overshadow the experience of the nerve injury; they should be free from current psychiatric or cognitive disorders; and they should be able to communicate in Swedish. Thirty participants in the former study were aged 12–20 years when the nerve injury occurred and was repaired. Two of these were suffering from a current psychiatric disorder and 20 participants were identified as eligible for the present study. The eligibility was decided by the results from the larger study [14] and defined by those who had experienced a considerable impact on their professional career, and/or on their education, and/or on leisure activities. Five declined participation due to lack of time or living too far away; fifteen accepted (twelve males and three females) and joined the present qualitative study in the year 2012. The median age when the nerve injury was sustained was 16 years (range 13–20) and the median follow-up time was 31 years (range 23–40). The dominant hand was affected in nine cases and there were nine participants with a median nerve injury, one with an ulnar nerve injury and five with both median and ulnar nerve injuries with a least one completely cut. Sural nerve grafts harvested from the lower leg were used in three participants. All participants completed several self-report questionnaires (Swedish version) before the interview; the DASH (Disability Arm Shoulder hand) [15] , the CISS (Cold Intolerance Symptom Severity) [16,17] and the VAS (Visual Analogue Scale) [18] for impact on education and leisure activities and finally the Sense of Coherence (SOC) condensed 13-item scale [19]. The SOC questionnaire is based on the salutogenetic theory introduced by Antonovsky more than 30 years ago [20] in which he claims that the way people view their life has a positive influence on health. The patient’s replies in the questionnaires reflect their disposition to see the world as comprehensible, manageable and meaningful [21].

The participants’ characteristics, including the clinical results, are presented in the larger study [14] and in Table 1 for comparison.

**Table 1:** Participant characteristics in the study (n=15)

**Age at nerve injury**	**16 (13–20)**
Gender: male/female (n)	12/3
Years of follow-up	31 (23–40)
Dominant hand (n), yes/no	9/6
Mechanism of injury (n)	
	
Cut by glass (window or bottle)	13
Cut by porcelain	1
Crush injury	1
Injured nerve (n)	
	
Median nerve	9
Ulnar nerve	1
Both nerves	5
Rosen score (0–3)	2.1 (0.4-2.5)
	25/ 75 percentile 1.7/ 2.3
DASH (0–100)	8 (0–61)
CISS (4–100)	41 (10–74)
Impact on profession yes/no (n)	7/8
VAS education (0–100)	76 (0–98)
VAS leisure (0–100)	52 (1–98)
SOC (13–91)	68 (52–89)

Values are medians (min-max if not specified as number (n) or percentile).

The participants are a heterogeneous group of patients from a previous study [14]. The total Rosen score is the sum of three different domains; sensory, motor and pain/ discomfort [27]. The maximum score is 3, which indicates a normal sensory and motor function without pain or discomfort. The cut-off for a pathological CISS score is 50 [17] and four participants had a pathological score. The impact on education and leisure activities was estimated with the use of VAS (Visual Analogue Scale) where 0 means no symptoms or problems and 100 indicate worst possible outcomes. We used the condensed 13-item version of the Sense of Coherence scale [19].

### Procedure and ethics

Written information was given by the first author emphasizing the aim and voluntary nature of the study. The last author then contacted patients and an interview time was set up with those who agreed to participate. Written consent was obtained in conjunction with the interview and patients were informed about how the data would be analyzed and were assured of confidentiality in the reporting. The study was conducted according to the ethical guidelines stated in the Helsinki Declaration and approved by the local ethics committee of Lund University (Dnr 2009/728). All interviews were performed and tape-recorded by the last author, in a quiet room at the clinic and lasted from 30-77 minutes (mean 45 minutes). The interview started with a repetition of the aim of the study. A semi-structured interview guide with open questions was then used and the participants were asked to describe the symptoms of their nerve injury, the consequences for their daily life, personal qualities and support from others. In addition, they were asked to describe strategies that facilitated adaptation and how symptoms and consequences had developed over time. Follow-up questions were asked such as: How did you experience that? How did you handle that? Can you describe that in more detail? A secretary transcribed all the interviews verbatim, marking nonverbal expressions and all transcripts were checked for accuracy by the last author. The first author did the translation from Swedish to English and the last authors then verified the translation.

### Data analysis

The text was read and reread by the first and last co-authors and a content analysis was performed [22]. The analysis started with a naive reading of each interview in order to gain a general impression of the content. Meaning units, that is words or phrases related to the aim of the study, were identified and coded with reference to the questions: “What is it about? What does it mean? What effect does it have?” [23]. The first and last authors discussed their impression of the text and compared their selected meaning units. Meaning units and codes that were similar in content were grouped together as categories. Similar statements within each category were analyzed critically, read and compared to achieve a reasonable interpretation. The categories were then discussed with the second author and adjustments were made to make sure that the categories covered all aspects. Finally, the categories were compared with the text and with each other. The second author read seven randomly selected interviews and reviewed the different codes and the categories. Concerning the authors’ pre-understanding, the first and second authors are experienced hand surgeons, the last author is an experienced occupational therapist specialized in hand rehabilitation. All three authors work in specialized unit. Both the second and the last authors are familiar with qualitative research methodology [24,25].

## Results

An overview of the main categories and the subcategories is presented in Table 2, including the participants’ experiences of symptoms related to the nerve injury, its consequences for daily life and the strategies and personal qualities important for adaptation. Furthermore, the participants described the information and support they would like to receive from the health care system.

**Table 2:** An overview of the main categories and the subcategories

**Main categories**	**Subcategories**	
Symptoms experienced and adaption strategies	Symptoms	Sensibility
		Pain/allodynia
		Dexterity
		Numbness
		Grip strength
		Cold sensitivity
		Secondary back/neck problems

	Adaptive strategies	Vision
		Non-injured hand/fingers
		Tricks
		Assistive devices/warm gloves/warm water
Emotional reactions to trauma and adaptation	Symptoms	Shock*
		Depressive symptoms
		Isolated*
		Bitterness
		Frustration
		Anger
		Grief
		Identity
		Ashamed
		Fear *

	Adaptive strategies	Social support
		Dissociation*
		Minimization
		Avoidance*
		Acceptance
		Hide/cover up*
Consequences for education and support provided	Consequences	Fell behind
		Grades affected
		Retake a school year
		Choose different education
		Not believed/no understanding

	Adaptive strategies	Assistive devices
		Information and support teachers/school mates
		Oral instead of written task/examination
Consequences for professional life and adaptation	Consequences	Choice of profession
		Obstructed the performance
		No support from employers and authorities

	Adaptive strategies	Realistic career plans
		Vision
		Avoidance
		Changed performance
		Develop new skills
Consequences for domestic life and adaptation	Consequences	Role as a parent and spouse
		Managing daily life

	Adaptive strategies	Open communication
		Ask for help
		Assistive devices
Consequences for leisure activities and adaptation	Consequences	Activities impossible/could still perform
	Adaptive strategies	Avoidance
		Develop new interests
Personal qualities		Persistence/ Endurance
		Positive attitude
		Problem-solver / Creative
		Competitiveness
		Purposefulness
		Avoid showing vulnerability
		Refusal to be a victim
		Enhance other capacities
Information and support wanted from the healthcare system		Oral and written information
		Help with assistive devices
		Help with insurance issues
		Psychosocial counselling
		Meet other patients

Consequences and adaptations strategies are presented as well as personal qualities and information and support wanted from the healthcare system. The asterisk represents symptoms related to post-traumatic stress disorder.

### Symptoms experienced and adaption strategies

A reduced *sensibility* in the injured hand was described, still present after three decades - “It feels like dental anaesthesia” or - “My hand feels dead, like a clump”. Certain participants described some improvement of the sensibility over time - “It took ten years” or - “It took twenty years before sensation came back”. It was important to use *vision* or the *non-injured hand/non-injured fingers* to compensate for the reduced sensation when performing tasks that require hand control - “I have to see what my fingers are doing”. Another participant with a median nerve injury said - “My little finger is mine for sensation”. A participant with a left-side injury explained - “I never put my keys in my left-hand pocket”. Others stated - “I have learned how to write with my other hand” or -“I have become more ambidextrous”. Different *tricks* were mentioned by the participants for managing difficulties in daily life such as - “I use my nail to feel the edge” or - “When I sew, I will soak my finger to be able to push the needle through”. *Assistive devices*, such as a pen or a potato peeler with a thickened grip, could enable participants to manage the challenges of daily life.

The reduced sensations led to an awareness of the risk of a new injury and many reported burning, cutting or squeezing their hands because of lack of sensation. However, there were participants who said that having sensibility was not as important for them as having strength - “I can do without sensibility”.

Remaining *pain* and *allodynia* over the surgical scar were also symptoms described during the interviews - “Like an electrical shock”. Other problems experienced were reduced *dexterity* and *numbness* in situations such as buttoning up a shirt or holding a pen and writing. While some participants said that it was difficult to know the amount of *grip strength* needed for a task, such as writing, others had no problems.

The symptoms described in connection with *cold sensitivity* were freezing more quickly, colour changes, stiffness and reduced dexterity. Pains, aches and dryness were other consequences. The symptoms had either decreased or increased over the years and were triggered by low temperatures, moist conditions or weather changes. Such symptoms were present even during summer time - “I cannot go on boat trips in summer time”. Strategies to relieve cold sensitivity were mentioned such as wearing thick warm gloves when exposed to cold - “I wear a glove even in summer time” or - “I will put my hand in lukewarm water”- to speed up the time needed for re-warming.

During the interviews there were reports of *secondary back* and *neck problems* related to the injured hand. The asymmetrical strain of trying to compensate for the reduced hand function had led to back pain and even sick leave in some cases. Since a few had undergone a nerve reconstructive procedure with a sural nerve graft harvested from the lower leg, there were also reports about donor site morbidity, such as pain/ache and reduced sensibility in the foot.

### Emotional reactions to trauma and adaptation

The participants gave very detailed descriptions of how the accidents occurred, despite the length of time that had passed. The trauma was a *shock* causing subsequent nightmares and flashbacks - “I can still wake up and hear the sound of broken glass”. However, some had amnesia about the event. Distorted thoughts about the injured hand were described, such as - “It was not mine; it was strange and disgusting…”

As they were teenagers when the injury occurred some stated it made them realize that they were not immortal. *Depressive symptoms,* such as feelings of sadness, darkness and hopelessness were mentioned. In addition, participants described living a passive life after the injury, *isolated* and withdrawn from social events. *Bitterness*, *frustration* and *anger* over the situation were mentioned during the interviews and a sense of *grief* over the hand and the life they had before the trauma was expressed. Their *identity* changed with the trauma and one participant said - “I became the injury”. Another participant still felt *ashame*d, 31 years later, for not telling the truth about how the injury occurred throughout the years. In the interviews, the participants described different strategies used to cope with the trauma experienced in the acute phase and later in life. Some participants described the *social support* from family and friends as very important for processing the trauma and stressed the importance of talking to others about it. Another strategy described was to try to *dissociate* oneself from the circumstances after the nerve injury. *Minimization*, comparing oneself with other hand-injured patients, was mentioned. In addition, comparisons with other disease groups, such as disabled people gave one perspective - “You have to play down the situation, it is only a hand”. Following the trauma and currently, an awareness of risks and a *fear* of a new injury existed - “I am still afraid of windows”. By *avoiding* certain situations, they tried to protect themselves and their family members. *Accepting* their limitations were important, as well as accepting the new appearance of the forearm - “You learn to live with your defects”. Another way of dealing with the new appearance was to *hide* or to *cover* the hand with scarves or bandages. One participant said - “For several years, I wore a thin bandage around my hand to hide it, but later I decided I was done with that…”

### Consequences for education and support provided

Being of school ages when the injury occurred created difficulties for the participants, according to their description. Hospitalization and time for rehabilitation led to absence and they *fell behind* with their schoolwork. Their *grades* were *affected* negatively, especially in more practical school subjects, such as drawing, crafts and physical training. Some had to *retake a school year* or *choose a different education* - “The ninth grade was completely ruined”. Another participant stated - “I was determined to become a physical education teacher, but no, it was impossible”. However, others stated that the injury had no influence on their education and that they were able to choose future occupations such as truck drivers and janitors - “My injury has not been permitted to limit my career choices”. No obvious associations were found between the consequences of a single nerve injury as opposed to injury to both the median and ulnar nerve, or to injuries affecting the dominant hand as opposed to the non-dominant hand. Several participants described how their teachers did *not believe* them when they said they had difficulties with writing for example. *Assistive devices* were scarcely used. During the interviews some mentioned that their teachers and schoolmates provided no support or had *no understanding*. However, others said that their friends in school did actually *help* them to perform difficult tasks. Another example of support received was that the head teacher *informed* all the other teachers about the participant’s nerve injury and suggested alternative tasks for the student, such as an *oral instead of a written task*.

### Consequences for professional life and adaptation

The nerve injury had influenced some of the participants’ *choice of profession* and some expressed sorrow at being unable to follow their chosen path. However, others felt that the injury constituted no hindrance. One person explained - “With my job as an ambulance driver I meet patients like myself…when they say that their lives are ruined, I am a living example of the opposite”. The participants advised future patients to talk to family and friends, teachers and guidance counsellors about their future *career plans* and to try to be *realistic* - “Maybe you cannot become the tennis player that you always dreamt about”. The hand function after the injury, with reduced grip strength, dexterity and sensibility *obstructed the performance* of certain work tasks. Various occupations were represented in the study group, such as electrician, plumber, janitor, truck driver, ambulance driver and teacher. Through using *vision* and changing the technique they used, several participants could compensate for reduced function by themselves. Examples given were - “I know what the bolt looks like so I can screw it on anyway” or - “I wrote every article by hand using paper and a pen. I then proofread it and rewrote it on the typewriter”. While some *avoided* difficult occupations, others continued, but *changed* their *performance pattern*. Certain participants described how they had altered their work tasks, *developed new skills* or patience with their performance - “The impossible just takes a little more time”. One participant worked as a chef, and had difficulties handling a knife and performing tasks that demand fine motor ability. However, this did not stop him from continuing in the same profession. Another participant had been in the military - “It takes a lot of time to assemble a weapon in the darkness”. Some experienced problems such as pain, numbness and reduced grip function when working outdoors, exposed to a cold and windy environment, which meant they needed a longer time to perform the work. Other described experiences of receiving *no support* and little understanding from *employers and authorities* after the injury. They felt that they often had to struggle against social insurance and employment offices.

### Consequences for domestic life and adaptation

Several situations were described where, in their *role as a parent*, the injury had had consequences, for example playing with their children and changing diapers - “When I walk with my child holding hands, if I use my healthy hand then I hold their hand, otherwise they hold my hand”. Concerns about family members injuring themselves were expressed, as well as admissions to being overprotective - “I get angry if my children are taking risks”. “The children are not allowed to be close to the glass window”. In their *role as a spouse*, intimacy was affected in various ways - “I never fondle with my injured hand” one participant stated. Another described - “Walking with my girlfriend holding hands, I had to choose the right side”. Furthermore, one participant stated - “In bed, I have to choose the right side; intimacy does not work so well, we are less spontaneous”. However, some of these problems could be avoided by open *communication* with their partners. *Managing daily life* could entail difficulties in peeling potatoes or handling cutlery in the kitchen. Fastening and unfastening a necklace or a shirt with buttons could be impossible and *asking for help* led to irritation. Participants described how using assistive devices, such as a pen or a potato peeler with a thickened grip, enabled them to manage.

### Consequences for leisure activities and adaptation

Several *activities* were reported as being *impossible* after the nerve injury, such as playing the guitar, tennis or table tennis. Numbness, a tendency to muscular cramp and tiredness in the hand were symptoms that affected performance. Various participants also *avoided* such activities as volleyball and soccer, because of the risk of the ball hitting the scar formation on the forearm. The participants said they *found new interests* and activities instead. While some reported difficulties with outdoor activities, such as fishing, hunting and playing hockey, because of sensitivity to cold, others experienced no problems with skiing or skating. Running and riding a motorbike were other *activities* that some *could still perform*. One participant had been one of the most promising athletes in the country in his sport but - “Because of the injury, I lost so much, I could not come back”.

### Personal qualities

All participants were asked which personal qualities had been important for them when dealing with life after the nerve injury. *Persistency* and *endurance* were frequently mentioned - “You have to be patient…never give up”. Participants said during the interviews that they had matured during the process of dealing with the consequences. A *positive attitude* towards obstacles, trying to see the possibilities instead, was also important - “You have to believe there is a solution for everything”. Participants described themselves as *problem-solvers*, imaginative and *creative*, having to adjust to new ways of performing different tasks. Characteristics such as *competitiveness* and *purposefulness* were also reported - “Nothing is going to stop me”. Some believed that they *avoided showing vulnerability* by being proud and not asking for help; others *refused to victimize* themselves. One statement explained how the nerve injury had *enhanced other capacities* - “At work, I will always turn down a written task, however I will always say yes to a verbal task”.

### Information and support wanted from the healthcare system

All participants expressed a desire for information and support from the health care system after the nerve injury. A need for *oral and written information* about the consequences of the nerve injury to be used in school or at work was stated. The participants importantly expressed the wish for clear and honest information about the expected consequences and prognosis, without eliminating hope. Other wishes were for help with *assistive devices* and *insurance issues*. Most participants were teenagers at the time of the nerve injury and sometimes did not understand the medical language used by adults. The importance of the healthcare staff involved in the treatment talking directly to the child or teenager, not only to their parents, was emphasized. Involving the relatives in the information and in the rehabilitation process could be a good support. *Psychosocial counselling* was introduced in our clinic in the middle of the 1970s and many described a wish for such support. It was emphasized that simple questions, like “how are you” or “how are you coping”, should be asked and that enough time should be given to listening to the answers. Taking part in the rehabilitation program was considered important and never giving up-“It is your choice”. *Meeting other patients* with severe hand injuries was also said to be important, especially if the other patient had got further on in their rehabilitation process.

## Discussion

**Figure 1:**
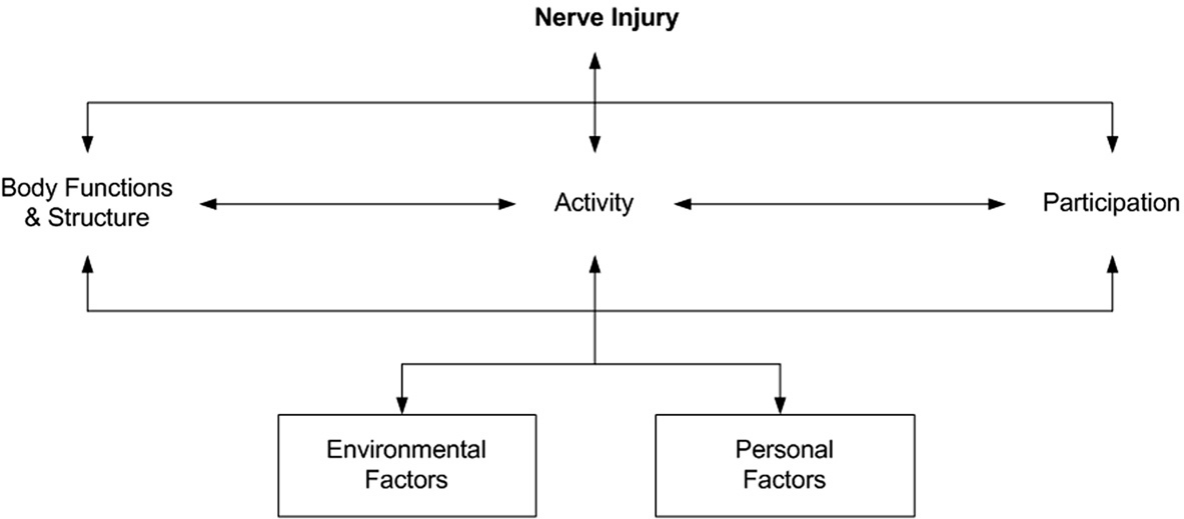
**Nerve injury- impact on functioning, disability and health (ICF).** The ICF is the WHO (World Health Organization) framework for measuring health and disability [26].

This study shows that a nerve injury in the upper extremity will have various consequences for the adolescent patient from a long-term perspective. Our participants experienced the commonly known functional deficits in the injured hand, as well as cold sensitivity. However, secondary problems, such as back problems, were also mentioned. The symptom severity, its consequences and adaptation to daily life in this group of patients reveal an impact on all levels outlined in the ICF (International Classification of Functioning, Disability and Health) [26], including activity limitation and participation restriction (Figure 1). The novel findings are the different emotional reactions to trauma and symptoms related to post-traumatic stress disorder described by the participants as well as how they managed to cope with such reactions. The participants stressed the need for support from the healthcare system, taking into consideration both physical and emotional needs. In addition, schoolteachers should be informed about such injuries, allowing them to provide help and understanding. Professional life and work performance changed completely for some while others simply changed their pattern of performance. Finally, something not clearly emphasized earlier, their life roles and the relations were affected.

The symptoms described as problematic by the study participants are in line with the different domains included in the Rosen score (sensory, motor, pain/discomfort) [27]. This further validates this outcome instrument on the level of body function [26]. However, the impact on the activity and participation level needs to be addressed using other outcome measures, sensitive to changes over time and valid for nerve injuries [1].

New findings were symptoms related to post-traumatic stress disorder (PTSD) such as subsequent nigthmares, flashbacks, avoidance and isolation. PTSD has previously been reported after acute hand injuries [7,9] and can have an impact on the outcome of the injury. However, there was no systematic screening for PTSD during the interviews in this study but this needs to be explored further in the future.

To learn more about the participants’ disposition to respond to stressful situations we used the condensed 13-item SOC questionnaire. The results reflect the SOC of the study participants three decades after the nerve injury (Table 1), where the median score of 68 indicates that the study participants had a high ability to comprehend, manage and find a meaning in their situation [19]. This is further supported by the diverse and abundant adaption strategies described during the interviews.

In our previous study, we asked the participants about the nerve injury’s impact on leisure activities and education [14]. The current study adds more information about how their schoolwork was affected and shows that some participants had to retake a school year or change their course. This needs to be considered when treating patients who are still students. In addition, we should involve adults who see such youngsters in other settings, inform them about the seriousness of the injury and ask them to be aware of the symptoms and consequences following the trauma [28]. Healthcare staff should also consider how they communicate with adolescent patients and their individual learning style, such as visual or auditory. Written educational brochures can reinforce oral information. By helping the patient to recall and understand we can enhance adherence to treatment and satisfaction [29,30].

The impact on engagement in leisure activities after nerve injuries should be explored and support given when needed. It might still be possible for the patients to engage in the activity if the**y** receive guidance on how to change their performance technique or are provided with assistive devices. The ability to perform meaningful activities and to maintain their roles in life are important for the patients’ quality of life [6,25,31]. The interviews described changes in life roles, as a parent and as a spouse, over the years. After the nerve injury, some participants felt dependent on practical help but could not accept the need for help from their children, as this would mean defeat. Interestingly, in the interviews, the participants described how intimacy with a partner could be affected by a nerve injury in the forearm. Not only will lack of sensation and dexterity lead to difficulties with intimacy, one also has to consider the new appearance of the hand and the patient’s perception of body image. Publications about sexual dysfunction after traumatic hand injuries are scarce [32]. These issues need more attention and should be further investigated to better help patients with hand injuries in general and nerve injuries in particular.

Coping strategies mentioned in this study are similar to those previously described after hand injuries [24,25,33]. Problem-based strategies [34], such as using vision or the non-injured digits or hand instead to compensate for the functional loss, were mentioned. Assistive devices, tricks or different ways of performing tasks and activities enabled them to master challenges in daily life. Enhancing other capacities or reorienting and finding other activities instead were also mentioned. Different emotion-based strategies [34] in the acute phase and later in life were described**.** Avoiding many questions from others by hiding or covering up the hand and accepting one’s limitations were ways of adapting to the situation, as well as comparing oneself with others in worse circumstances and minimizing one’s own situation. Social support from others was important for processing the trauma and rehabilitation groups where patients can share their experiences and meet others who have got further in their rehabilitation process can play an important role.

Patients with a nerve injury today receive postoperative care that is quite different from that given to the participants in this study. It is believed that cerebral reorganisation plays a crucial role after a peripheral nerve injury [12] and early (phase 1) and late (phase 2) sensory relearning has been introduced. Studies indicate that sensory relearning can improve the functional outcome after a nerve injury [35] and early onset is advocated [2]. In addition, patient motivation is also important for the outcome after a nerve injury [36,37] and early referral to mental health professionals can reduce psychological morbidity after a severe hand injury [6,8,13]. Dealing with the psychological distress and individual perception of the injury might improve patient adherence to rehabilitation and enhance the outcome of treatment [38].

Content analysis was used to study an individual process occurring over long period of time and the trustworthiness of the findings was evaluated by establishing credibility, dependability and confirmability [22]. Credibility was achieved by using purposive sampling and as shown in Table 1, the participants’ results show a wide variation in the different parameters. The small proportion of women corresponds to the typical pattern found in the clinic and to hand injuries in general [39,40]. Dependability was achieved by the co-authors reading and coding all the interviews independently and by in-depth discussions, analysis and interpretation of the text together. Representative quotations from all participants are presented in the text in order to increase the trustworthiness of the statements. Confirming and clarifying information during the interviews ensured confirmability. The risk of over-interpretation was reduced by focusing consistently on the text throughout the analysis. We used pre-defined open questions, but the transferability in this study is limited since the participants represent a small study group. However, using in-depth interviews allows the patient to spontaneously reveal problems and other information not reported if more structured self-report questionnaires are used.

## Conclusions

This study shows that treating patients with nerve injuries at forearm level is complex and requires attention to other aspects as well as to repair the damaged nerve. In the clinical practise today it can help us to provide the patient with honest and realistic information about what to expect after a nerve injury at forearm level. Healthcare professionals can promote different coping mechanisms in order to facilitate the daily living for the injured patients. In addition, emotional reactions to trauma and symptoms related to post-traumatic stress disorder should be identified and taken care of immediately. With a better understanding, we might be able to improve the outcome after a nerve injury and the patient satisfaction in the future.

## Competing interests

The authors declare that they have no competing interests.

## Authors’ contributions

AC, LBD and IKC participated in the design of the study. IKC conducted the interviews. AC and IKC read and analyzed the data from the interviews. The categories were discussed with LBD and adjustments were made to make sure that the categories covered all aspects. LBD read seven randomly selected interviews and reviewed the different codes and the categories. AC wrote the manuscript and LBD and IKC revised and approved the manuscript. All authors read and approved the final manuscript.

## Pre-publication history

The pre-publication history for this paper can be accessed here:

http://www.biomedcentral.com/1471-2474/14/252/prepub
